# MRBEE: A novel bias-corrected multivariable Mendelian Randomization method

**DOI:** 10.21203/rs.3.rs-2464632/v1

**Published:** 2023-02-01

**Authors:** Noah Lorincz-Comi, Yihe Yang, Gen Li, Xiaofeng Zhu

**Affiliations:** 1 Department of Population and Quantitative Health Sciences, Case Western Reserve University.

**Keywords:** mendelian randomization, genetic epidemiology, statistical genetics, complex disease

## Abstract

Mendelian Randomization (MR) has been widely applied to infer causality of exposures on outcomes in the genome wide association (GWAS) era. Existing approaches are often subject to biases from multiple sources including weak instruments, sample overlap, and measurement error. We introduce MRBEE, a computationally efficient multivariable MR method that can correct for all known biases simultaneously, which is demonstrated in theory, simulations, and real data analysis. In comparison, all existing MR methods are biased. In two independent real data analyses, we observed that the causal effect of BMI on coronary artery disease risk is completely mediated by blood pressure, and that existing MR methods drastically underestimate the causal effect of cannabis use disorder on schizophrenia risk compared to MRBEE. We demonstrate that MRBEE can be a useful tool in studying causality between multiple risk factors and a disease outcome, especially as more GWAS summary statistics are being made publicly available.

## 1 Introduction

Mendelian randomization (MR) is a widely used statistical tool that uses genetic variants as instrumental variables (IVs) to estimate causal relationships between exposures and outcomes [1–3]. Originally developed for application in individual level data [2], many MR methods can also be applied to summary-level statistics obtained from genome-wide association studies (GWASs) and have therefore become increasingly popular to infer causality of disease risk factors [3], identify biological drug targets [4], and causal effects of genes on phenotypes [5]. Despite its popularity and flexibility, valid causal inference from MR relies heavily on certain assumptions which may not typically be satisfied or testable in practice [6]. The three primary MR assumptions are that the genetic IVs are (i) strongly associated with the exposures, (ii) not directly associated with the outcome conditional on the exposures, and (iii) not associated with any confounders of the exposure-outcome relationships. In the literature, violations of these assumptions are respectively referred to as weak instrument [10], uncorrelated horizontal pleiotropy (UHP) [3], and correlated horizontal pleiotropy (CHP) [7] biases.

These assumptions may be routinely violated in practice and so many methods intended to provide unbiased causal estimates in these cases have been developed. UHP bias is addressed by methods such as MRPRESSO/IMRP [20, 22], MR-Median [23], MR-Robust [24], and MR-Mix [15]. Methods designed specifically to additionally address CHP bias include CAUSE [7], MR-CUE [16], and MR-Corr [19], which each assume the IV set is a mixture of CHP and otherwise valid IVs. CHP can be considered to result from the omission of additional exposures in MR, but these methods cannot accommodate multiple exposures and so must make complex statistical adjustments based on additional assumptions that may not be satisfied in practice.

**Fig. 1:**
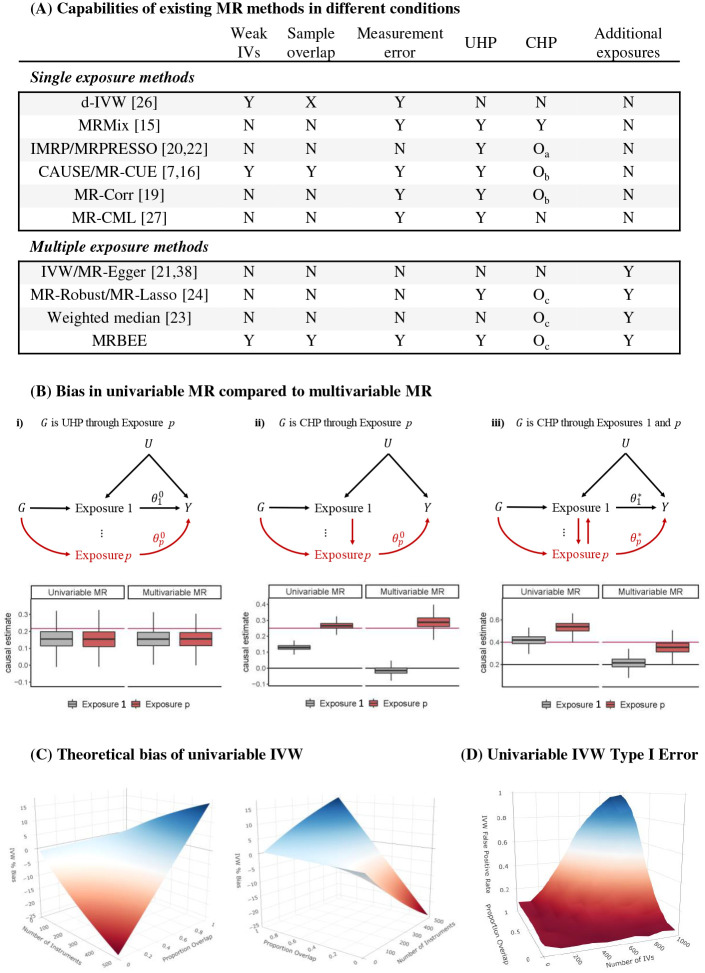
**(A)**: Bias addressed by currently available MR methods. ‘N’: cannot address. ‘Y’: can fully address. To be ‘Y’, ‘O_a_’ requires that all CHP IVs resemble outliers, ‘O_b_’ requires proportion of CHP IVs to be <0.10, and ‘O_c_’ requires that all moderately genetically correlated exposures with CHP effects are included in MR or that all CHP resembles outliers. Each available MR method can only address a subset of all bias known to affect causal estimation. No univariable MR method can estimate direct causal effects, while no existing multivariable MR method to estimate direct causal effects can do so without bias besides MRBEE. **(B)**: Situations in which univariable MR with IVW cannot reliably estimate direct causal effects. Multivariable IVW can more reliably estimate direct causal effects, but still suffers from large bias. Horizontal gray and red lines respectively indicate true direct effects of exposure 1 and *p*. Boxplots are causal estimates from simulation with true relationships represented by the directed acyclic graphs. Boxplot center lines are medians and boxes are formed by the lower and upper 25% quantiles. **(C)**: Relative bias (bias divided by true causal effect times 100) using Equation 1 is displayed for different numbers of IVs and overlap proportions when the exposure and outcome GWAS sample sizes are 30k.

Besides the biases mentioned above, two additional biases include measurement error [8] and sample overlap [9]. Measurement error refers to the difference between the true genetic association of an IV with a phenotype and its estimate from GWAS that is used in MR. Sample overlap refers to the overlapping of individuals in exposure and outcome GWAS used in MR. These biases can drastically inflate or deflate causal estimates and Type I/II error rates of causal effect hypothesis testing. Most MR methods completely ignore bias from measurement error and sample overlap, and no MR method can robustly address all sources of bias simultaneously (Figure 1A).

Of the methods that can address these two additional biases (i.e., CAUSE, MR-CUE), still none can robustly address CHP bias because they assume the proportion of CHP IVs is small (e.g., <10%). However, there is substantial evidence that many phenotypes are at least moderately genetically correlated [12, 13] and so may share more causal variants than just a small proportion. A more robust, simpler, and computationally efficient way to reduce or even eliminate CHP bias is to include multiple genetically correlated exposures in MR simultaneously (‘multivariable MR’), therefore making the CHP bias disappear automatically. This would also provide additional epidemiological inference regarding total, mediated, and direct causal effects. However, very few multivariable MR methods exist and they are each vulnerable to additional biases from weak instruments, sample overlap, and measurement error.

We propose the computationally efficient MRBEE (MR with Bias-corrected Estimating Equations), a multivariable MR method, and demonstrate in simulations and application to real data that only MRBEE can estimate total and direct causal effects without bias in a range of real-world conditions compared to all other MR methods we studied. We further point out that the popular F-statistics for measuring instrument strength [10, 25] are not reliable bias-detection tools in many cases. We demonstrate that weak instrument and measurement error biases are widely present in MR and encourage the use of bias-correcting causal estimators instead of bias-detecting tools such as the F-statistics. We finally apply MRBEE to two independent real data analyses, first estimating direct causal effects of cardiometabolic risk factors on coronary artery disease risk in two populations and second estimating direct causal effects of modifiable and non-modifiable risk factors for schizophrenia and bipolar disorder.

## 2 Results

### 2.1 Bias in MR

In multivariable MR, the direct causal effects of multiple exposures on an outcome phenotype are estimated with a set of *m* independent IVs that have evidence of association with at least one of the *p* exposures. Let ***θ*** be the *p*-length vector of causal effects of *p* exposures on the outcome, ***α*** be the *m*-length vector of true standardized effects of the *m* IVs on the outcome, and **B** be the *m* × *p* matrix of true standardized effects on the *p* exposures. Let ${\hat{\beta }}_{j}$ and ${\hat{\alpha }}_{j}$ be the estimated effect sizes of the *j*th IV on the *p* exposures and the outcome from exposure and outcome GWAS, respectively. Then ${\hat{\beta }}_{j}=\beta_{j}+w_{\beta_{j}}$ and ${\hat{\alpha }}_{j}=\alpha_{j}+w_{\alpha_{j}}$, where $w_{\beta_{j}}$ and $w_{\alpha_{j}}$ are the corresponding measurement errors for the exposures and outcomes due to sampling error (i.e., finite sample sizes) in GWAS. In Methods, we show the bias of current multivariable IVW MR approaches can be written as the product of two terms:

(1) $$\text{bias}\left({\hat{\theta }}_{\text{IVW}}\right)=\underset{\text{Weak instruments bias}}{\underset{︸}{{\left\{\frac{\Sigma_{\beta \beta }}{m}+\frac{1}{m}\overset{m}{\underset{j=1}{\sum }}\Sigma_{w_{\beta_{j}}{\text{w}}_{\beta_{j}}}\right\}}^{-1}}}\underset{\text{Sample overlap \& Measurement error}}{\underset{︸}{\frac{1}{m}\left\{\overset{m}{\underset{j=1}{\sum }}\sigma_{w_{\beta_{j}}w_{\alpha_{j}}}-\Sigma_{w_{\beta_{j}}w_{\beta_{j}}}\theta \right\}}},$$

where **Σ**_*ββ*_ is the *p*×*p* matrix of genetic covariance between the exposures using the *m* IVs, $\Sigma_{w_{\beta_{j}}w_{\beta_{j}}}=\text{Cov}\left(w_{\beta_{\text{j}}}\right)$, and $\sigma_{w_{\beta_{j}}w_{\alpha_{j}}}=\text{Cov}\left(w_{\beta_{j}},w_{\alpha_{j}}\right)$. The first term in Equation 1 represents bias from weak instruments because *m*^−1^**Σ**_*ββ*_ represents the average instrument strength, where it can be observed that each diagonal element in **Σ**_*ββ*_ represents the SNP heritability explained by the *m* IVs for each exposure. When there is sample overlap between an exposure and the outcome in GWAS, ${\hat{\beta }}_{j}$ and ${\hat{\alpha }}_{j}$ are correlated and $\;\sigma_{w_{\beta_{j}}w_{\alpha_{j}}}$ is nonzero [18]. The term $\Sigma_{w_{\beta_{j}}w_{\beta_{j}}}\theta$ represents the contribution of the measurement error variance (which is inversely proportion to the sample sizes of exposure GWAS [49]) for the exposures unless there are no causal effects of the exposures on the outcome. It is clear from Equation 1 that measurement error biases and bias from sample overlap are working in opposite directions.

These sources of bias are independent of UHP and CHP biases because the values in Equation 1 are completely determined by GWAS sample size, overlap proportions, true phenotypic correlations, and causal effects. These biases can go in any direction when multiple exposures are included in MR, may affect the power of horizontal pleiotropy testing as in MRPRESSO [22] and IMRP [20], Type I/II error rates of causal effect hypothesis testing, and will increase as the number of IVs increases (Figure 1C). As the number of IVs increases, the IV set on average becomes weaker, thereby enhancing biases from measurement error and sample overlap. When there is only one exposure, the bias term is simplified to

(2) $$\text{bias}\left({\hat{\theta }}_{\text{IVW}}\right)=\left(\frac{m}{n_{1}h_{X}^{2}+m\sigma_{X}^{2}}\right)\left(\frac{n_{0}\sigma_{XY}}{n_{2}}-\sigma_{X}^{2}\theta \right),$$

where *n*_1_ and *n*_2_ are the respective exposure and outcome GWAS sample sizes, *n*_0_ is the number of overlapping GWAS participants, $h_{X}^{2}$ is the SNP heritability explained by the *m* IVs, $\sigma_{X}^{2}$ is the phenotypic variance of the exposure, and *σ*_*XY*_ is the phenotypic covariance between the exposure and outcome (see **Supplement**).

Figure 1A lists the currently available MR methods and their capability to deal with various sources of bias. Existing MR methods cannot simultaneously address all known sources of bias. Including multiple genetically correlated exposures in MR may reduce CHP bias from omitted exposures, but will not necessarily reduce biases from weak instruments, sample overlap, or measurement error (Figure 1B). In our simulations, we observed that sample overlap can make the false positive rate for testing no causal effect of the exposure on outcome approach 100% even if GWAS sample sizes are reasonably large (~30k) (Figure 1D).

### 2.2 MR using Bias-corrected Estimating Equations (MRBEE)

We propose MRBEE (MR using Bias-corrected Estimating Equations) to correct for weak instrument, sample overlap, and measurement error biases while estimating direct causal effects of multiple exposures. We provide an additional adjustment for horizontal pleiotropy bias which is introduced later. Assume all GWAS estimates have been standardized (see, e.g., [15]) and multivariable IVW uses the following score function

(3) $$S_{\text{IVW}}(\theta )=\frac{-\partial ∥\hat{\alpha }-\hat{B}\theta {∥}_{2}^{2}}{2\partial \theta },$$

which does not have expectation 0 and therefore is responsible for the bias in Equation 1. The bias in Equation 3 is only eliminated asymptotically as GWAS sample sizes approach infinity. MRBEE uses the bias-corrected score function:

(4) $$S_{\text{MRBEE}}(\theta )=S_{\text{IVW}}(\theta )-\left(\overset{m}{\underset{j=1}{\sum }}\sigma_{w_{\beta_{j}}w_{\alpha_{j}}}-\Sigma_{w_{\beta_{j}}w_{\beta_{j}}}\theta \right),$$

which, in the absence of UHP and CHP, is asymptotically equivalent to maximizing a likelihood function that accounts for measurement error in the GWAS estimates (as in, e.g., MR-CUE [16]). The terms $\sigma_{W_{\beta_{j}}w_{\alpha_{j}}}$ and ${\text{Σ}}_{W_{\beta_{j}}W_{\beta_{j}}}$ are estimated using methods in [18, 50] (also used by MR-Corr [19], MR-CUE [16], and CAUSE [7]; see **Supplement** and [49]). Briefly, these methods estimate the correlation matrix for exposure and outcome GWAS estimates using all overlapping non-significant (e.g., P>0.05) SNPs in the exposure and outcome GWAS. This estimated correlation matrix is then converted to SNP-specific covariance matrices (which contains elements $\sigma_{w_{\beta_{j}}w_{\alpha_{j}}}$ and ${\text{Σ}}_{w_{\beta_{j}}w_{\beta_{j}}}$) using standard error estimates from GWAS. The full derivation and asymptotic properties of MRBEE are available in Methods and more extensively in [49] and the **Supplement**.

In addition to addressing UHP/CHP bias by including multiple exposures in MR, MRBEE can also identify specific IVs with evidence of horizontal pleiotropy and remove them from causal estimation. In many cases, specific IVs with UHP/CHP evidence will behave like outliers in regression. MRBEE uses a multivariate generalization of the IMRP algorithm [20] to identify such outliers and remove them using a new statistic *S*_pleio_. We also introduce the statistic *Q*_pleio_ to detect global unbalanced horizontal pleiotropy in the set of IVs which could bias causal estimates (see Methods). *Q*_pleio_ is a multivariate generalization of the popular MR-Egger intercept test [38].

**Fig. 2:**
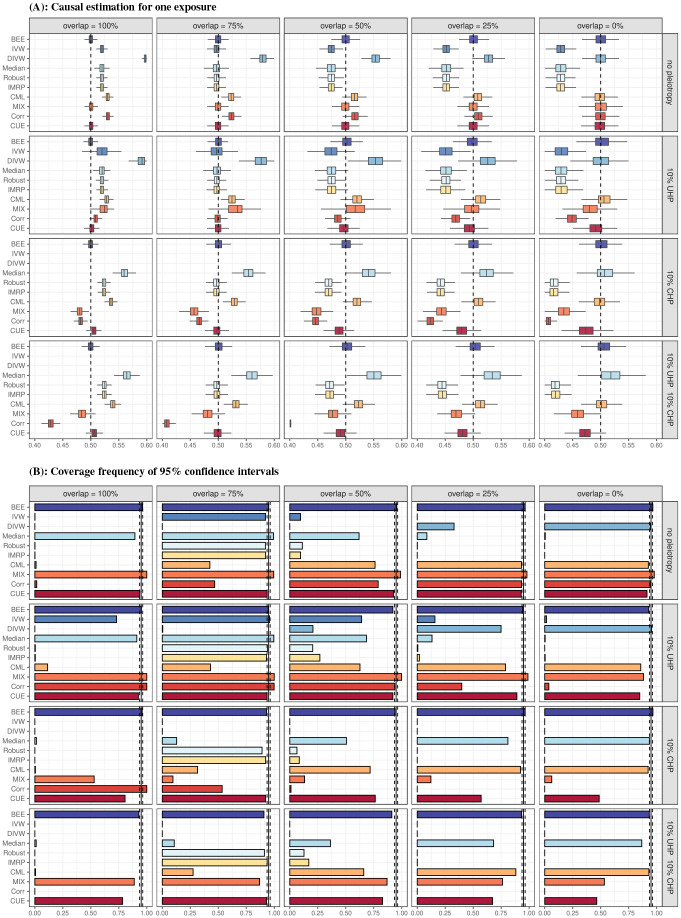
**(A)** Biases of causal effect estimation in simulation with one exposure with true total causal effect ***θ*** =0.5 (indicated by the vertical dashed line) and there are 1,000 IVs. Sample sizes of exposure and outcome GWAS were both 20k, phenotypic correlation was 0.5 between the random errors in the exposure and outcome, and exposure/outcome SNP heritability was 0.3/0.15. “10% UHP” means that 10% of IVs were randomly selected to have UHP effects with variance 4/m, “10% CHP” means that 10% of IVs were randomly selected to have CHP effects with variance 4/m, and “10% UHP 10% CHP” means that 10% of IVs were randomly selected to have UHP effects with variance 4/m and an alternative 10% of IVs were randomly selected to have CHP effects with variance 4/m. To make UHP and CHP resemble outliers, we generated UHP and CHP only in the lower and upper 5% tail regions of the corresponding normal distribution. **(B)** 95% confidence interval coverage frequencies for simulation with settings described in in caption for panel **A**.

Figure 2 demonstrates that MRBEE is the only MR method that can estimate the total causal effect of a single exposure without bias as UHP, sample overlap, GWAS sample sizes, and weak instrument bias varies compared to IVW [21], dIVW [26], weighted median [23], MR-Robust [24], IMRP [20], MR-CML [27], MRMix [15], MR-Corr [19], and MR-CUE [16] methods. MRBEE is therefore the only method of these that has controlled coverage frequencies (Type I error is also controlled, see Supplementary Section S3). MRBEE can also unbiasedly estimate true causal effects when only a small proportion of exposure variance is explained by the IVs (see Supplementary Section S1.1) since MRBEE corrects weak instrument bias. The traditional IVW method is biased in this case [10].

**Fig. 3:**
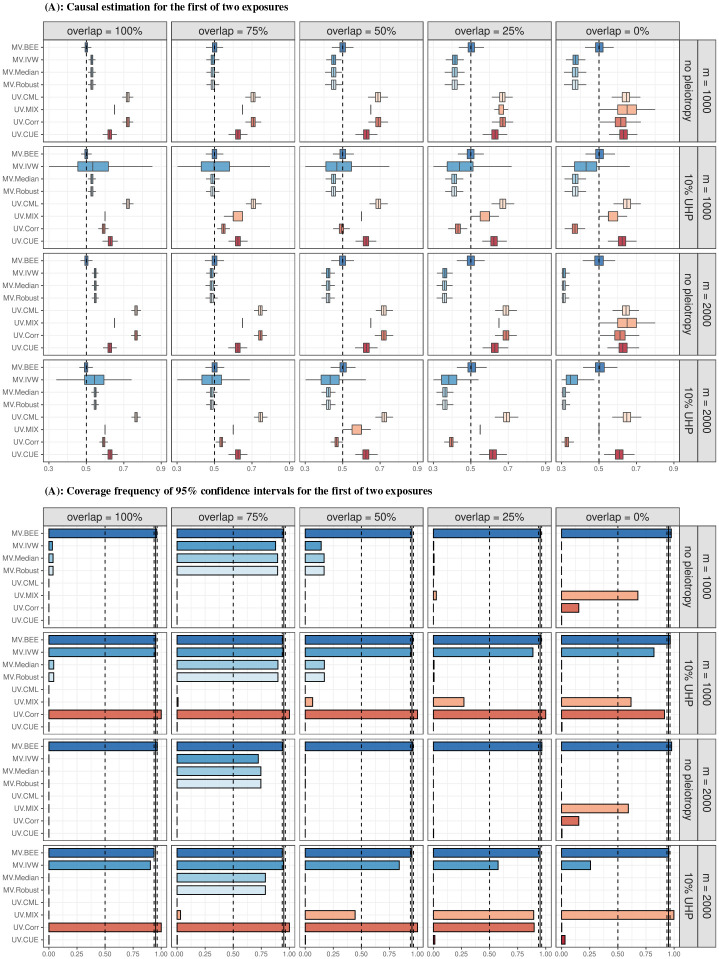
**(A)**: Bias in causal estimation of the direct effect for the first of two true and genetically correlated exposures and one outcome. The true causal effect is indicated by the vertical dashed line (0.5). MR methods that could only include exposure 1 in MR have ‘UV.’ prefixes; multivariable methods that included both exposures have ‘MV.’ prefixes. We simulated two exposures with nonzero causal effects on a single outcome by randomly drawing GWAS summary statistics for GWAS samples of size 20k, phenotypic and genetic correlations of 0.5 between all phenotypes, and exposure/outcome heritability of 0.30/0.15. ‘10% UHP’ means that 10% of IVs had UHP effects with variance 16/m. CHP is automatically introduced in univariable methods (UV.CML, UV.MIX, UV.CORR, UV.CUE) via the omitted exposure (see Supplementary Section S1.1.1 for full settings). **(B)**: Proportions of simulations in which the estimated 95% confidence interval of the causal estimate contained the true direct effect of exposure 1 (0.5).

Figure 3A demonstrates that, comapared to the alternative methods included in Figure 2, MRBEE is the only MR method that can estimate direct causal effects without bias in the presence of CHP. Multivariable MR methods (i.e., weighted median, IVW, MR-Robust) are generally less biased than univariable MR methods (i.e., MR-CUE, MR-CML, MR-Corr, MR-Mix), but still they cannot consistently estimate direct causal effects because of uncontrolled biases from weak instruments, measurement error, and sample overlap. Since every other MR method except MRBEE is biased, their coverage proportions are generally low. For example, the coverage proportion for MR-CML is less than 1% in every case in simulations where horizontal pleiotropy, sample overlap, and the number of IVs varied (Figure 3B), which may lead to incorrect causal inference in practice. MRBEE was the only MR method to obtain optimal coverage frequencies in all simulation settings. The univariable MR-Corr often has coverage proportions around or equal to 1 but it also has large standard errors.

#### 2.2.1 Genome-wide application of *S*_pleio_

**Fig. 4:**
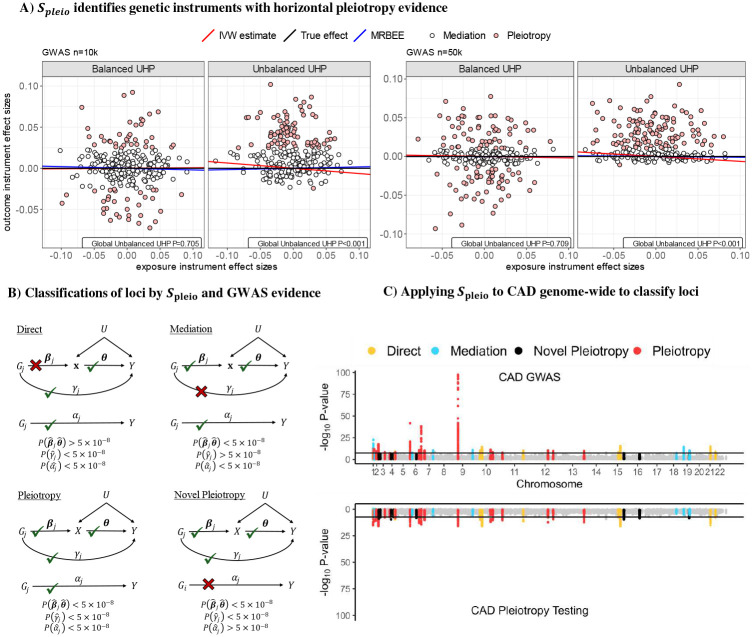
**(A)**: Demonstration of how horizontal pleiotropy IVs are identified in IMRBEE. A global test for balance is performed using statistic *Q*_pleio_ in Equation 22. An IV-specific test for horizontal pleiotropy is performed using statistic *S*_pleio_ in Equation 19. IVs represented by red points had a significant *S*_pleio_ statistic and are considered invalid; white points do not and are considered valid. **(B)**: Classifications of genomic loci by evidence from original GWAS and genome-wide pleiotropy testing using *S*_pleio_. Classifications are based on P-values [denoted as P(·)] for testing null hypotheses of equality with 0 for a given parameter in practice. **(C)**: Results from real data analysis, the methods and results for which are more completely described in the Methods and Results sections. MR exposures are height, BMI, uric acid, HDL, LDL, triglycerides, systolic blood pressure, HbA1c, and hemoglobin. The outcome is coronary artery disease (CAD). Classifications are made based on which of the four directed acyclic graphs in **Panel B** each locus follows.

The horizontal pleiotropy test statistic *S*_pleio_ can also be applied genome-wide to search for genomic loci with evidence of horizontal pleiotropy. The genome-wide application of univariable versions of *S*_pleio_ has previously successfully identified novel genomic regions undetected by standard GWAS testing [17]. When multiple exposures are included in MR, *S*_pleio_ can also be used to infer pathways of genetic association with a phenotype. That is, *S*_pleio_ can identify genomic regions whose association with a phenotype are completely mediated by a set of exposures. Figure 4B shows the four types of inference that can be made with *S*_pleio_ and Figure 4C demonstrates how *S*_pleio_ was applied to real data in the study of coronary artery disease (CAD) in Europeans with a set of nine exposures (see below).

### 2.3 Real Data Analyses

We performed two independent analyses of real data to demonstrate how MRBEE works and compares to existing MR methods in practice. We first estimated direct causal effects of 9 cardiometabolic risk factors on CAD risk in East Asian (EAS) and European (EUR) populations (Real Data Analysis 1). We next estimated direct causal effects of 7 known risk factors for schizophrenia (SCZ) or bipolar disorder (BP) in a European population (Real Data Analysis 2). We compared causal estimates from MRBEE to IVW* (defined below), MR-Robust (equivalent to MR-Lasso), and multivariable weighted median methods since these are the only multivariable MR methods that exist. IVW* refers to the multivariable IVW causal estimates after removing specific IVs with significant *S*_pleio_ statistics at a FDR-adjusted (for the number of IVs) Type I error rate of 5% using Benjamini-Hochberg [28] in the MRBEE procedure.

#### 2.3.1 Real Data Analysis 1 (Coronary Artery Disease)

Univariable MR results suggested nonzero causal effects of all exposures on CAD in EAS or EUR populations. However, there was widespread evidence of unbalanced horizontal pleiotropy as indicated by large differences in causal estimates between UHP-adjusted and UHP-näıve MR methods. For example, the causal effect of DBP on CAD in EAS was estimated to be 2.03 (OR; P=2.8×10^−11^) using IMRP but only 1.43 (P=0.140) using MR-Egger, where estimates from these two methods differ only by how UHP is addressed. Full univariable MR results are presented in the **Supplement**.

**Fig. 5:**
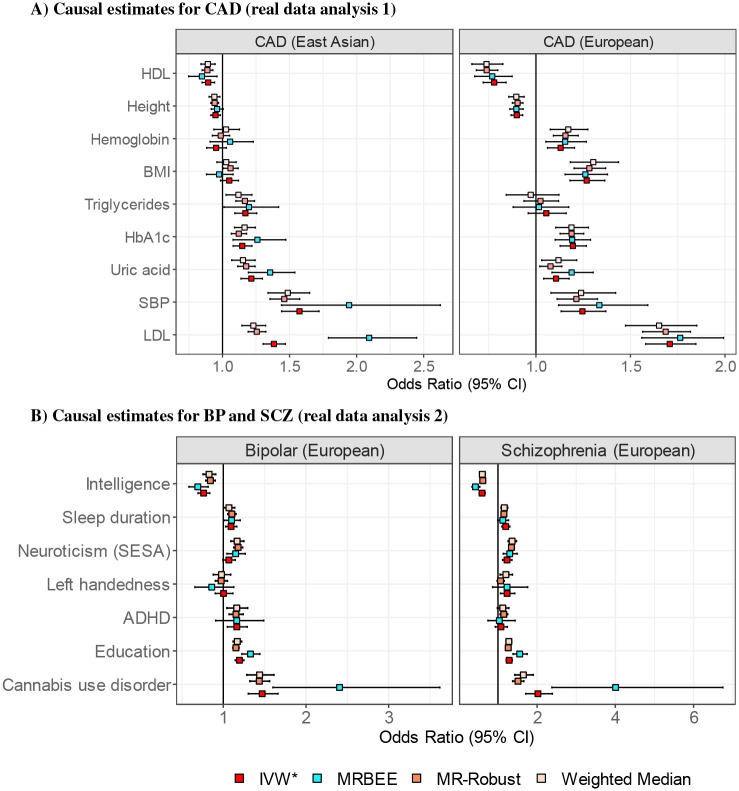
Direct causal estimates from multivariable MR are obtained from IVs whose selection is described in Methods. ‘IVW*’ represents the multivariable IVW estimator after removing specific IVs with evidence of horizontal pleiotropy using *S*_pleio_ in IMRBEE, adjusted for multiple testing using FDR. ‘IVW*’ is therefore more robust against UHP than IVW. We found no evidence of unbalanced horizontal pleiotropy in any analyses (see Results).

Multivariable MR estimates (see Figure 5A) using MRBEE were generally consistent between EAS and EUR populations. All 9 exposures had evidence of nonzero causal effect on CAD in EAS or EUR in population-specific testing. IVW*, MR-Robust, and weighted median generally had similar causal estimates. LDL had the strongest causal effect estimate in both EAS (MRBEE OR=2.09; P=1.1×10^−20^) and EUR (OR=1.76, P=4.1×10^−20^), which was undetected for EUR in [42] because of bias. Unlike in [42], we included HDL in multivariable MR and found negative nonzero effects on CAD in EAS (OR=0.85, P=0.011) and EUR (OR=0.77, P=3.7×10^−5^). In EAS, all other multivariable MR methods drastically underestimated the direct causal effect of LDL on CAD. For example, IVW* and MR-Robust produced odds ratio estimates of 1.39 (P=2.2×10^−25^) and 1.46 (P=1.2×10^−22^). The direct causal effect of SBP on CAD in EAS was similarly underestimated by IVW* compared to MRBEE (MRBEE OR=2.29, P=1.6×10^−11^ vs IVW* OR=1.56, P=5.0×10^−23^).

In EAS, the total causal effect of BMI on CAD (IMRBEE OR=1.44, =2.0×10^−25^) was completely mediated by SBP (P=0.220 for total mediation; see **Supplement**). Evidence of nonzero direct causal effect of BMI on CAD in EUR may be explained by the GWAS data used for SBP. In EUR, the SBP GWAS included BMI as a covariate and so SBP could not statistically act as a mediator for BMI in multivariable MR with CAD. The BMI result displayed in Figure 5A therefore reflects the effect of BMI on CAD through all other exposures except SBP. This phenomenon – that including one exposure as a covariate in the GWAS for another can preclude consistent direct causal effect estimation in multivariable MR – is confirmed in simulation (see **Supplement**). We draw attention to this issue here because it so far has not been considered in the multivariable MR literature but must be in order to make correct epidemiological inference.

**Fig. 6:**
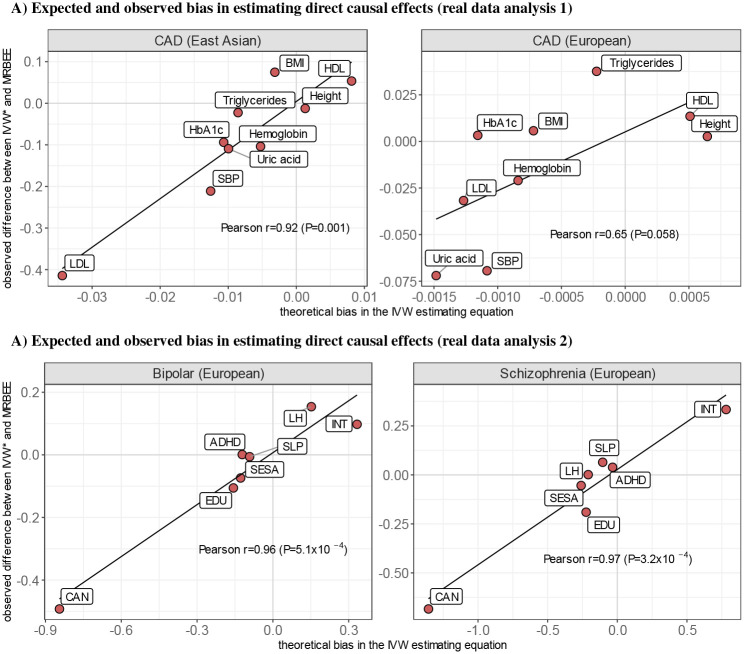
The x-axes represent theoretical bias in the direct causal effect estimates of IVW* (horizontal pleiotropy-robust IVW; see Figure 5 legend, **Results**), which was calculated using the expectation of Equation 1 with the plugged-in MRBEE direct causal estimates. Y-axes are the observed difference between the IVW* and MRBEE direct causal estimates. Pearson’s r values represent the linear correlation between values on the x- and y-axes. Corresponding P-values are for testing the null hypothesis that r=0.

Differences between MRBEE and IVW* causal estimates were strongly correlated with the bias in Equation 1 due to weak instruments, sample overlap, and measurement error (EAS Pearson r=0.92, P=4.6×10^−4^; EUR r=0.65, P=0.058; Figure 6A). Since there was no evidence of unbalanced horizontal pleiotropy (EAS P=0.528 for a test of balance using *Q*_pleio_, see Methods; EUR P=0.231), this suggested that differences between IVW* and MRBEE causal estimates were due to uncontrolled bias in IVW*. Since causal estimates made by IVW* were generally very similar to those made by MR-Robust and weighted median methods, a similar interpretation can be made for them, too.

#### 2.3.2 Real Data Analysis 2 (Schizophrenia and Bipolar Disorder)

Univariable MR results suggested nonzero total causal effects of cannabis use disorder (CUD), Attention-Deficit/Hyperactivity Disorder (ADHD), left handedness, neuroticism, sleep duration, intelligence, and education on BP or SCZ. Consistent with [29] but not [30, 31], we found a strong negative causal effect of left handedness on BP risk (IMRBEE OR=0.70, P=8.9×10^−34^), which is of opposite sign for SCZ (OR=1.36, P=6.2×10^−24^). Whether this is the result of inherent bias in univariable MR cannot be confirmed so full univariable results are reserved for the **Supplement**.

Multivariable MR with MRBEE and exposures CUD, ADHD, left handedness, neuroticism, sleep duration, intelligence, and education identified nonzero causal effects for all exposures on BP and/or SCZ except ADHD and left handedness in outcome-specific testing. IVW*, MR-Robust, and weighted median MR methods generally produced very similar causal estimates. IVW* drastically underestimated the direct causal effect of CUD on SCZ, where IVW* and MRBEE respectively produced odds ratio estimates of 2.02 (P=2.2×10^−16^) and 4.00 (P=1.8×10^−7^), the latter of which is most consistent with the literature (i.e., OR for CUD association is 3.90, 95% CI: 2.84–5.34 in [34]). IVW* additionally underestimated causal effects for education, intelligence, left handedness, and neuroticism compared to MRBEE for SCZ and BP (see Figure 5B). CUD had the largest causal effect on BP and SCZ, explaining as much as 16% and 39% of the genetic variance in BP and SCZ, respectively (see Methods).

Differences between IVW* and MRBEE causal estimates were almost perfectly correlated with the bias in Equation 1 due to weak instruments, sample overlap, and measurement error (Figure 6B; BP Pearson r=0.96, P=5.1×10^−4^; SCZ r=0.97, P=3.2×10^−4^). Since there was no evidence of unbalanced horizontal pleiotropy (global P=0.625 for joint test of balance for BP or SCZ using *Q*_pleio_), this suggested that MRBEE appropriately adjusted for these sources of bias while the IVW*, MR-Robust, and weighted median methods did not.

Follow-up testing using Equation 23 in Methods indicated that all exposures except left handedness and ADHD had nonzero direct causal effects on BP or SCZ (see Methods). These 7 exposures on average have stronger causal effects on SCZ than BP (P=2.5×10^−5^), though the effect of sleep duration (P=0.698) and ADHD (P=0.415) on BP and SCZ may be similar (P-values correspond to a test of difference). There was no evidence that modifiable risk factors including CUD, sleep duration, and education had in total greater direct causal effects than the largely non-modifiable risk factors (all other exposures) on both BP (P=0.265 for difference) and SCZ (P=0.267).

#### 2.3.3 Genome-wide Horizontal Pleiotropy Testing

We finally performed genome-wide horizontal pleiotropy testing for CAD, SCZ and BP using *S*_pleio_, for which a summary of the results are displayed in Table 1.

Of the 65 and 39 loci (1Mb, r^2^<0.01, P<5×10^−8^) identified by standard GWAS testing for CAD in EAS and EUR, respectively, horizontal pleiotropy testing correspondingly identified 27 ($\lambda_{GC}^{\text{pleio }}=1.08$ vs $\lambda_{GC}^{\text{GWAS }}=1.16$) and 41 ($\lambda_{GC}^{\text{pleio }}=1.01\;$ vs $\lambda_{GC}^{\text{GWAS }}=1.00$ in GWAS) loci. In EUR, nine loci that were detected in horizontal pleiotropy testing were not detected in the original CAD GWAS. Seven of these were replicated (P<0.05 for lead SNP) in an independent CAD GWAS in EUR (UKBB; [51]), all of which could only be detected in a larger CAD GWAS [32]. In EUR and EAS, we respectively identified only 10 and 18 loci that are directly associated (i.e., not indirectly through the MR exposures) with CAD and 19 (EUR) and 5 (EAS) with evidence of simultaneous association (i.e., horizontal pleiotropy) with the MR exposures and CAD conditional on the exposures.

We also identified one locus for SCZ using *S*_pleio_ genome-wide that was not discovered in the original SCZ GWAS (i.e., GWAS P=9.3×10^−3^, *S*_pleio_ P=3.5×10^−8^). This locus maps to the *ATXN2L* gene which is known to be associated with intelligence [35], brain morphology [36], and autoimmune disease [37]. Of the 152 and 44 loci identified for SCZ and BP in the original GWAS, 151 (98%) and 42 (95%) were not associated at the level of genome-wide significance respectively with SCZ and BP through pathways that did not include CUD, intelligence, education, sleep duration, or neuroticism (see Methods). This suggests that a large portion of the heritability of these two traits may be due to genetic associations with their causal risk factors and not directly with the psychiatric disorders themselves.

## 3 Discussion

Our study provides strong evidence that the existing univariable and multivariable MR approaches are vulnerable to biases from weak instruments, measurement error, UHP, CHP, sample overlap, and omitted exposures. One suggested solution to this problem that is currently being heavily practiced in the literature is to use multiple MR methods and appraise the evidence in aggregate more highly than evidence from any one method alone [33]. Our applications of MRBEE to real data demonstrated that multiple MR methods can be biased in similar ways, rendering any aggregated inference from multiple biased methods no less subject to mistake than inference from any one method alone. In contrast, the multivariable MRBEE we developed here is generally robust to the above biases and can be a useful tool in practice [49].

We demonstrated the practical utility of MRBEE in two independent applications to the study of CAD in (i) EAS and EUR and (ii) schizophrenia (SCZ) and bipolar disorder (BP). MRBEE was able to explain and correct inconsistent causal estimates made by existing MR methods. Causal risk factors were generally consistent for CAD between EAS and EUR and between SCZ and BP in EUR, where any differences between MRBEE estimates and those made by alternative methods were the results of uncontrolled bias in other methods. For example, the causal estimate of LDL on CAD in EAS was expected to have 55.3% downward bias from Equation 1 and indeed the horizontal pleiotropy-robust IVW causal estimate was 55.7% smaller than the MRBEE estimate (Figure 5A). In Real Data Analysis 1 with CAD, we observed that the total causal effect of BMI on CAD was completely mediated by blood pressure and partially by uric acid in EAS, though the GWAS data in EUR precluded testing of this kind. In Real Data Analysis 2 with schizophrenia (SCZ) and bipolar (BP), we observed that cannabis use disorder (CUD) has very large direct causal effects on SCZ and BP risk (consistent with the literature [34], but that existing MR methods drastically underestimate the sizes of these effects.

We finally introduced a multivariable horizontal pleiotropy test (using statistic *S*_pleio_) that, when applied genome-wide, identified the epidemiological pathways through which many genomic loci were associated with CAD in EAS and EUR, SCZ, and BP. The majority of known genetic associations with all disease endpoints were non-direct (Table 1), suggesting that a large portion of the heritability of these complex traits may be conferred indirectly through their causal risk factors. This test also identified 9 novel loci (undetected in standard GWAS) for CAD in EUR – seven of which were replicated in UKBB – and one for SCZ in EUR, for which no adequate independent replication data exists. This method of pleiotropy testing using *S*_pleio_ is therefore a valuable tool both for gaining better insight into how genetic risk of disease is conferred and in detecting new risk loci.

In conclusion, single-exposure MR is inherently limited in its ability to reduce bias or provide precise epidemiological inference, but univariable MR methods and their application have so far dominated the literature compared to multivariable analyses. We developed multivariable MRBEE to reduce known biases in MR and estimate direct causal effects of multiple exposures in robust way. MRBEE can be a useful tool in studying causality between risk factors and disease outcomes as more large GWAS summary statistics are made publicly available.

## 4 Method

### 4.1 Measurement error and its bias

More complete asymptotic properties for many quantities introduced below are found in [49]. Mendelian Randomization (MR) uses single-nucleotide polymorphisms (SNPs) as instrumental variables (IVs) to estimate causal effects of select exposures on an outcome. Let **g** represent the *m*-length vector of IVs, **x** be the *p*-length vector of exposures, *Y* be the outcome, ***θ*** be the *p*-length vector of causal effects of **x** on *Y*, **B** be the *m* × *p* matrix of true associations betweeen **g** and **x**, ***α*** be the *m* × 1 vector of true associations between **g** and *Y*, ***γ*** be the *m*-length vector of associations between **g** and *Y* conditional on **x**, and *U* be effects of confounders of the relationships between **x** and *Y*. Then the following models hold:

(5) $$x=B^{⊤}g+U+e_{x},$$

(6) $$Y=\theta^{⊤}x+\gamma^{⊤}g+U+e_{Y}=g^{⊤}\alpha +e_{Y}^{*}.$$

As a result, it can be obtained that

(7) α=Bθ+γ,

where it is generally assumed in MR that for the set of *m* IVs ***γ*** = **0** (i.e., there is no horizontal pleiotropy in the IV set). In practice, we only have estimates of ***α*** and **B** from GWAS, which we denote as $\hat{\alpha }$ and $\hat{B}$. Since these are estimated from GWAS, we can state the following measurement error models for ***α*** and $B={\left(\beta_{j}\right)}_{j=1}^{m}$:

(8) $${\hat{\alpha }}_{j}=\alpha_{j}+w_{j},$$

(9) $${\hat{\beta }}_{j}=\beta_{j}+w_{j},$$

where

$$\left(\begin{array}{c} {\hat{\alpha }}_{j}\\ {\hat{\beta }}_{j} \end{array}\right)\sim N\left(\left[\begin{array}{c} \theta^{⊤}\beta_{j}\\ \beta_{j} \end{array}\right],\left[\begin{array}{cc} \sigma_{w_{\alpha_{j}}}^{2} & \sigma_{w_{\beta_{j}}w_{\alpha_{j}}}^{⊤}\\ \sigma_{w_{\beta_{j}}w_{\alpha_{j}}} & \Sigma_{w_{\beta_{j}}w_{\beta_{j}}} \end{array}\right]\right).$$

The IVW estimating equation, denoted as *S*_*IVW*_(***θ***), is an equation that when set equal to **0** is used to find the IVW causal estimator. If the expectation of *S*_*IVW*_(***θ***) is not **0**, then the IVW causal estimates are biased. The bias in *S*_*IVW*_(***θ***) is the following:

(10) $$\text{E}\left[{\text{S}}_{\text{IVW}}(\theta )-0\right]=\text{E}\left[\overset{\text{m}}{\underset{\text{j}=1}{\sum }}\beta_{\text{j}}\left({\hat{\alpha }}_{\text{j}}-\beta_{\text{j}}^{⊤}\theta \right)\right]=\overset{\text{m}}{\underset{\text{j}=1}{\sum }}\sigma_{{\text{w}}_{\beta_{\text{j}}}{\text{w}}_{\alpha_{\text{j}}}}-\Sigma_{w_{\beta_{\text{j}}}{\text{w}}_{\beta_{\text{j}}}}\theta .$$

Using the Taylor series expansion of *S*_*IVW*_(***θ***), it can be shown that

(11) $$\sqrt{m}\left({\hat{\theta }}_{IVW}-\theta^{0}\right)=\sqrt{m}{\left[-\frac{\partial }{m\partial \theta^{0}}S_{IVW}\left(\theta^{0}\right)\right]}^{-1}m^{-1}S_{IVW}\left(\theta^{0}\right),$$

where ***θ***^0^ represents true causal effects and $\hat{\theta }$ represents the IVW causal estimates. It is shown in the **Supplement** and [49] that the expectation of Equation 11, which is equal to the bias of the IVW estimator, is equal to the quantity in Equation 1.

The measurement errors in Equation 8 and 9 represent estimation error due to sampling variability in GWAS and have the stated properties only if GWAS estimates are unbiased, which they generally are. In practice, different genetic variants may have different genotyping rates, especially between cohorts that may be pooled together in meta-analyses, so the measurement error variances between different IVs may differ. We treat the measurement error variance-covariance matrices as fixed since the corresponding correlated matrices can typically be estimated from approximately one million genetic variants using the methods in [18, 50]. Briefly, these methods involve first estimating the correlation between GWAS estimates (${\hat{\alpha }}_{j}$, ${\hat{\beta }}_{j}$), then using the standard error estimates from GWAS (treated as fixed) to calculate the corresponding variance-covariance matrices.

### 4.2 Unbiasedness of MRBEE

We propose MRBEE to correct for the bias in *S*_*IVW*_(***θ***) which will automatically eliminate that bias in the IVW causal estimates (i.e., in Equation 1). We use the following bias-corrected estimating equations:

(12) $$\psi (\theta )=\overset{m}{\underset{j=1}{\sum }}\psi_{j}(\theta )=\overset{m}{\underset{j=1}{\sum }}{\hat{\beta }}_{j}\left({\hat{\alpha }}_{j}-{\hat{\beta }}_{j}^{⊤}\theta \right)-\sigma_{w_{\beta_{j}}w_{\alpha_{j}}}+\Sigma_{w_{\beta_{j}}{\text{w}}_{\beta_{j}}}\theta .$$

Let ${\hat{\theta }}_{\text{MRBEE}}=\hat{\theta }$ be the estimate for ***θ*** produced from *ψ*(***θ***). It is shown in the **Supplement** that

(13) $${\hat{\theta }}_{\text{MRBEE}}\overset{D}{\to }N\left(\theta ,m^{-1}F^{-1}VF^{-1}\right),$$

(14) $$F=m^{-1}\left(B^{⊤}B-\overset{m}{\underset{j=1}{\sum }}\Sigma_{w_{\beta_{j}}w_{\beta_{j}}}\right)=\text{E}\left(\frac{-\partial \psi (\theta )}{m\partial \theta }\right),$$

(15) $$V=m^{-1}\overset{m}{\underset{j=1}{\sum }}\text{E}\left[\psi_{j}(\hat{\theta })\psi_{j}{\left(\hat{\theta }\right)}^{⊤}\right].$$

Equation 13 simply states that causal estimates made using MRBEE will asymptotically follow a normal distribution (see [49] for convergence properties), which is useful for statistical inference. In practice, we can use the following sample estimates of **F** and **V**:

(16) $$\hat{F}=m^{-1}\left({\hat{B}}^{⊤}\hat{B}-\overset{m}{\underset{j=1}{\sum }}\Sigma_{w_{\beta_{j}}w_{\beta_{j}}}\right),$$

(17) $$\hat{V}=m^{-2}\psi (\hat{\theta })\psi {\left(\hat{\theta }\right)}^{⊤},$$

which can be shown to respectively converge in probability to **F** and **V** in [49]. We demonstrate in simulations in the **Supplement** that $m^{-1}{\hat{F}}^{-1}\hat{V}{\hat{F}}^{-1}$ approximates *m*^−1^**F**^−1^**VF**^−1^ well.

### 4.3 Detecting IVs with horizontal pleiotropy

In the following, we assume all CHP is either included explicitly via additional exposures or behaves as outliers in regression. Unbalanced horizontal pleiotropy can make causal estimates inconsistent. The null hypothesis for the *j*th IV of no horizontal pleiotropy is the following:

(18) $$H_{0j}:\alpha_{j}-\beta_{j}^{⊤}\theta =\gamma_{j}=0\text{ vs }H_{1j}:\gamma_{j}\neq 0.\;$$

We propose the following statistic to test this null hypothesis for the *j*th genetic variant which may or may not be used as an IV in MR:

(19) $$S_{\text{pleio }}^{\left(j\right)}={\left({\hat{\alpha }}_{j}-{\hat{\beta }}_{j}^{⊤}\hat{\theta }\right)}^{⊤}{\hat{\Upsilon }}_{j}^{-1}\left({\hat{\alpha }}_{j}-{\hat{\beta }}_{j}^{⊤}\hat{\theta }\right)\overset{D}{\to }\chi^{2}(1)$$

under *H*_0*j*_ where ${\hat{\Upsilon }}_{j}$ is any consistent estimator for the variance of ${\hat{\alpha }}_{j}-{\hat{\beta }}_{j}^{⊤}\hat{\theta }$. The only assumption here is that ${\hat{\alpha }}_{j}-{\hat{\beta }}_{j}^{⊤}\hat{\theta }$ is asymptotically normal, which it is as proven in [49] and stated above in Equation 13. In practice, we can estimate Υ_*j*_ using the delta method as

(20) $${\hat{\Upsilon }}_{j}=\sigma_{\alpha_{j}}^{2}+{\hat{\theta }}^{⊤}\Sigma_{w_{\beta_{j}}w_{\beta_{j}}}\hat{\theta }+{\hat{\beta }}_{j}^{⊤}{\hat{F}}^{-1}\hat{V}{\hat{F}}^{-1}{\hat{\beta }}_{j}-2{\hat{\theta }}^{⊤}\sigma_{w_{\beta_{j}}w_{\alpha_{j}}},$$

which is shown to converge in probability to the true value **Υ**_*j*_ in [49]. This test can be performed for every genetic variant available or only for those specific genetic variants used as IVs in MR.

Similar to the MR-Egger [38] adjustment, we can perform a test for global unbalanced horizontal pleiotropy by adding intercept terms to MRBEE causal estimation. Let ***θ***_0_ denote the intercept term in multivariable MRBEE causal estimation. It is shown in the **Supplement** that testing the null hypothesis that ***θ***_0_ = 0 is equivalent to testing the null hypothesis that the mean of the IV-specific horizontally pleiotropic effects is 0. More explicitly we can test

(21) $$H_{0}:\theta_{0}=m^{-1}\overset{m}{\underset{j=1}{\sum }}\gamma_{j}=0\text{ vs }H_{1}:m^{-1}\overset{m}{\underset{j=1}{\sum }}\gamma_{j}\neq 0$$

to identify the presence of global unbalanced horizontal pleiotropy. Let θ* = (*θ*_0_, ***θ***^⊤^)^⊤^ and **Λ** be the asymptotic variance of ${\hat{\theta }}^{*}$ to which $\hat{\Lambda }$ from Equations 16 and 17 converges in probability. We can test *H*_0_ in Equation 21 using the statistic

(22) $$Q_{\text{pleio }}=\frac{s^{⊤}\theta^{*}\theta^{*T}s}{s^{⊤}\hat{\Lambda }s}\overset{D}{\to }\chi^{2}(1),$$

where **s** is a (*p* + 1)×1 vector with leading element 1 and remaining elements 0 that is used only to extract the intercept-relevant terms from ${\hat{\theta }}^{*}$ and $\hat{\Lambda }$. In practice, $\hat{\Lambda }$ is estimated using the same method as in Equations 16 and 17, but now where intercept-relevant quantities are included in $\hat{B}$, $\Sigma_{w_{\beta_{j}}w_{\beta_{j}}}$, and $\sigma_{w_{\beta_{j}}}w_{\alpha_{j}}$. The result in Equation 22 is proven in the **Supplement**. In practice, if *Q*_pleio_ is sufficiently large, there is evidence of global unbalanced horizontal pleiotropy that can bias causal esitmation and specific IVs with evidence of large *S*_pleio_ values should be removed. The IMRP method of removing specific IVs with large *S*_pleio_ values is more completely described in [20], but basically follows the steps in Table 2 below.

### 4.4 Joint testing of causal effects

In **Real Data Analysis 2** with schizophrenia and bipolar disorder, we performed joint and contrast testing of causal effects for the outcome phenotypes in the following way. Denote ${\hat{\Theta }}_{\left(2\right)}$ as the (7 + 1) × 2 matrix of causal effect estimates, where the first column of ${\hat{\Theta }}_{\left(2\right)}$ corresponds to the exposure effects on SCZ and the second on BP. Let ${\hat{\Lambda }}_{\left(2\right)}$ denote the corresponding variance-covariance matrix of $\text{vec}\left({\hat{\Theta }}_{\left(2\right)}\right)$. Since the same IVs were used for SCZ and BP, elements of **Θ**_(2)_ and **Λ**_(2)_ were estimated using a single multivariate MRBEE model. We wished to test the general linear hypotheses of the following form:

(23) $$H_{0}:C^{⊤}\Theta_{\left(2\right)}L=\mu \text{ vs }H_{1}:C^{⊤}\Theta_{\left(2\right)}L\neq \mu ,$$

where **C** and **L** were respectively *g* × *h* and *s* × *l* fixed design matrices with elements chosen for specific hypotheses as indicated in the Results section. Let **H** = (**L** ⊗ **C**)^⊤^ and $\hat{\vartheta }=\text{vec}\left({\hat{\Theta }}_{\left(2\right)}\right)$ and we tested different *H*_0_ in Equation 23 using the statistic

(24) $${\left(H\hat{\vartheta }\right)}^{⊤}{\left(H\hat{\Lambda }H^{⊤}\right)}^{-1}(H\hat{\vartheta })\overset{D}{\to }\chi^{2}(hl),$$

The convergence statement in Equation 24 is proven in the **Supplement**.

### 4.5 Simulation settings

Unless stated otherwise, all simulations used simulated GWAS summary statistics that were drawn from normal distributions specified by parameters that depended on GWAS sample size, sample overlap proportions, phenotypic correlations, and true causal effects that are fully described in Section 3 of the **Supplement**. True causal effects were determined by genetic correlations, exposure and outcome SNP heritability, and any specified horizontally pleiotropic effects. All phenotypic and genotypic variances were equal to 1 so the true variance of any GWAS estimate was the inverse of the respective GWAS sample size. Pleiotropic effects were randomly drawn with specified variances by allowing the final term in Equation 7 (***γ***) to be nonzero for a defined proportion of IVs.

### 4.6 Real Data Analysis 1: Coronary Artery Disease

#### 4.6.1 GWAS data

More complete descriptions of the GWAS data used are available in the **Supplement**. EAS exposure GWAS data were provided exclusively by Biobank Japan [39]. EAS CAD GWAS data were provided by [40] (n=212k). EUR exposure GWAS data were provided by a range of consortia as described in the **Supplement**. EUR CAD GWAS data were provided by the CARDIoGRAM consortium (n=184k) [41]. Cardiometabolic risk factors used in multivariable MR for CAD included serum lipids (HDL, LDL, triglycerides), BMI, systolic blood pressure, uric acid, height, HbA1c, and hemoglobin. Hematocrit, diastolic blood pressure, and red blood cell count were excluded from multivariable MR because of high correlations (>0.75) in IV estimates with other exposures. Full details of the GWAS data used are available in Section 4 of the **Supplement**.

#### 4.6.2 MR instrumental variables

We performed univariable (single exposure) and multivariable (multiple exposure) MR and generally followed the methods of [42] in selecting IVs. Candidate IVs in univariable MR analysis were associated (P<5×10^−8^) with the exposure in a within-phenotype and between-ancestry fixed-effects GWAS meta-analysis of EAS- and EUR-specific GWAS, had the same sign in the EAS and EUR GWAS, and had at least P<0.05 in both GWAS. We then selected only independent (r^2^<0.01 in a 1Mb window using PLINK v1.9; [43]) SNPs from this set using ancestry-specific linkage disequilibrium (LD) reference panels from 1000 Genomes Phase 3 [44]. In [42], the authors then removed IVs associated (P< 5×10^−8^) with any of 36 other phenotypes, which is a step we did not complete so as to not unnecessarily reduce the exposure variance explained by our IVs. Indeed, our IVs explained more exposure variances than those in [42] (see **Supplement**). Only ancestry-specific GWAS estimates were used in ancestry-specific MR. For multivariable MR in each ancestry group, all IVs from univariable MR were initially considered, then filtered to only include those SNPs that were independent (r^2^<0.01 in a 1Mb window using ancestry-specific LD reference panels from 1000 Genomes [44]). As mentioned in the Results, there was no evidence of unbalanced horizontal pleiotropy in EAS or EUR using *Q*_pleio_ (Equation 22) so we did not remove any IVs using *S*_pleio_ in IMRBEE but did include an intercept term in MRBEE. This resulted in 3,097 IVs used in EAS and 2,821 in EUR. Differences in IV counts differed between EAS and EUR only because of the differences in availability of certain SNPs in the EAS and EUR GWASs. Results from alternative selections of the IVs used in MR are available in the **Supplement** and are consistent with those presented in the Results section. All GWAS estimates were standardized following the methods in [15].

#### 4.6.3 Genome-wide horizontal pleiotropy testing

Since all exposures had evidence of nonzero causal effect on CAD in either EAS or EUR, all 9 exposures were included in genome-wide horizontal pleiotropy testing using *S*_pleio_. Genome-wide pleiotropy testing with *S*_pleio_ was performed separately in EAS and EUR. Lead SNPs in independent loci (r^2^<0.01, 1Mb, P<5×10^−8^) identified in the original CAD GWAS were compared to the horizontally pleiotropic effects of the same lead SNPs to produce the results in Table 1.

### 4.7 Real Data Analysis 2: Schizophrenia and Bipolar

#### 4.7.1 GWAS data

More complete descriptions of all GWAS data used in MR are available in the **Supplement**. Exposures for SCZ and BP included cannabis used disorder (CUD), left handedness (LH), insomnia, sleep duration, BMI, systolic and diastolic blood pressure, Attention-Deficit/Hyperactivity Disorder (ADHD), gestational duration, intelligence, neuroticism (sensitivity to environmental stress and adversity; SESA), cerebral volume, and glutamate and dopmaine neurological measurements. Only CUD, LH, ADHD, sleep duration, education, intelligence, and neuroticism were considered in multivariable MR because only these had evidence of nonzero total causal effect on either BP or SCZ in univariable MR using MRBEE (see **Supplement**). All GWAS data were from studies in strictly EUR individuals, which are from a range of consortia (see **Supplement**). Exposure GWAS sample sizes ranged from 55k for ADHD [47] to 1.7M for LH [48]. SCZ GWAS data were from meta-analysis performed using data from the Psychiatric Genomics Consortium [45] on 130k EUR individuals (53k cases). BP GWAS data were from [46] that had a total sample size of 413k (42k cases) EUR individuals, where the outcome phenotype was defined as either lifetime Bipolar I or II disorder.

#### 4.7.2 MR instrumental variables

Many exposure GWAS could only identify a few (e.g., 2 for CUD) SNPs that reached genome-wide significance (P<5×10^−8^) and were independent (r^2^<0.01, 1Mb window) with other SNPs using a EUR LD reference panel from 1000G [44]. We therefore considered all independent SNPs with exposure GWAS P<5×10^−5^ in univariable MR analysis. We then excluded 3 IVs whose minor allele frequency differed by more than 0.10 from all other exposures. All independent IVs from univariable MR were initially considered for multivariable MR. We then filtered this list of 3,346 IVs to only include those associated with the 7 exposures selected for multivariable MR at P<5×10^−8^ in a 7-degree of freedom joint test for association with the 7 selected MR exposures. We did not remove any IVs with large *S*_pleio_ statistics because tests of global unbalanced horizontal pleiotropy using *Q*_pleio_ (Equation 22) did not suggest any imbalance (see Results) for BP or SCZ. This resulted in 504 IVs that were used in multivariable MR. IV estimates were standardized by standard error and not by the methods in [15] because the GWAS sample size for left handedness was considerably larger (n=1.7 million) than those for the remaining exposures. Since the standardization in [15] is based explicitly on sample size, we standardized only by standard error for the results presented in the Results section to ensure the scale of causal effect estimates was not heavily influenced by exposure GWAS sample size. Results from standardization using the methods in [15] are presented in the **Supplement** and the inferences are consistent with the results presented in the Results section.

#### 4.7.3 Genome-wide horizontal pleiotropy testing

Since ADHD and left handedness each had no evidence of direct causal on either BP or SCZ in multivariable MR, we excluded them from genome-wide horizontal pleiotropy testing with *S*_pleio_. Including left handedness and ADHD in genome-wide pleiotropy testing would have effectively only increased the variance term used in *S*_pleio_ (see Equation 19) and not otherwise affect the inferences we could make. Genome-wide testing with *S*_pleio_ was performed separately for SCZ and BP. Lead SNPs in independent loci (r^2^<0.01, 1Mb, P<5×10^−8^) in the original SCZ and BP GWAS were used for comparisons with results from genome-wide horizontal pleiotropy testing to produce the results in Table 1.

### 4.8 Software

The software used to perform all simulations and analyze the real data used above is available at github.com/noahlorinczcomi/MRBEE and http://hal.case.edu/~xxz10/zhu-web/ The software contains all functions needed to use MRBEE and perform all their associated tests in practice.

## Figures and Tables

**Table 1:** Counts of specific locus types from genome-wide horizontal pleiotropy testing

	Mediation	Direct	Pleiotropy	Novel^1^	Novel Replicated Genes
CAD (EUR)	9 (19%)	10 (21%)	19 (41%)	9 (19%)	*FN1,FGD5,PRDM8,FGF5,FURIN,CFDP1,AXL*
CAD (EAS)	37 (62%)	18 (30%)	5 (8%)	0 (0%)	NA
SCZ (EUR)	151 (9s%)	1 (1%)	0 (0%)	1 (1%)	*ATXN2L* ^2^
BP (EUR)	42 (95%)	2 (5%)	0 (0%)	0 (0%)	NA
·					

Values represent counts of loci identified in genome-wide testing for the trait. Percentages within each row sum to 100 and represent the percentage of GWAS loci that can be classified into each of the four categories. Loci are determined with the following parameters: r^2^<0.01, 1Mb, P<5×10^−8^. Categories are determined using the algorithm in Figure 4B

a detected by horizontal pleiotropy testing but not GWAS signal within +−1Mb of the lead pleiotropy SNP was discovered

b Insufficient independent GWAS data exist to replicate the association of this gene with SCZ

**Table 2:** IMRP algorithm with MRBEE

**(1)**	**Initialize**: Estimate ***θ*** using MRBEE and all IVs
**(2)**	**Pleiotropy test**: Remove all IVs with *S*_pleio_ > *ξ*
**(3)**	**Causal estimation**: Estimate ***θ*** using MRBEE
**(4)**	**Iteration**: If ${\left\|{\hat{\theta }}_{k}-{\hat{\theta }}_{k-1}\right\|}_{1}>\tau$ at iteration *k*, repeat steps 2–3; else, stop: $\hat{\theta }={\hat{\theta }}_{k}$

*ξ* is chosen by the researcher, but is generally a quantile of the *χ*^2^(1) distribution that is corrected for the number of IVs used, *p* is the number of exposures, and *τ* is also selected by the researcher and should be relatively small (e.g., <0.001) to ensure that true convergence can be achieved. All MRBEE estimates used in this iterative procedure can include intercept terms.

## Data Availability

All GWAS data used for the analyses were retrieved from publicly available repositories whose online locations are presented in Supplementary Tables 1S and 6S. Genomic loci detected in either the original genome-wide association studies or in genome-wide horizontal pleiotropy testing in Real Data Analyses 1 & 2 are available at github.com/noahlorinczcomi/MRBEE.
